# ZNF367-induced transcriptional activation of KIF15 accelerates the progression of breast cancer

**DOI:** 10.7150/ijbs.44204

**Published:** 2020-05-16

**Authors:** Huijuan Zeng, Tianfu Li, Duanyang Zhai, Jiong Bi, Xiaying Kuang, Sihong Lu, Zhen Shan, Ying Lin

**Affiliations:** 1 Breast Disease Center, The First Affiliated Hospital of Sun Yat-sen University, Guangzhou 510080, China.; 2 Laboratory of Surgery, The First Affiliated Hospital, Sun Yat-Sen University, Guangzhou, 510080, China.

**Keywords:** ZNF367, KIF15, breast cancer, cell cycle

## Abstract

Breast cancer (BC) is one of the most common female cancers, and its incidence has been increasing in recent years. Although treatments are continuously improving, the prognosis of patients in the advanced stage is still unsatisfactory. Thus, an in-depth understanding of its molecular mechanisms is necessary for curing breast cancer. KIF15 is a tetrameric spindle motor which can regulate mitosis in cellular process and exert the crucial functions in several cancers. The purpose of our research was to investigate the functions of KIF15 in breast cancer. We tested the expression of KIF15 in breast cancer tissues and the survival rate of breast cancer patients with high or low level of KIF15 through TCGA data. What's more, western blot and immunohistochemistry assay were utilized to evaluate the protein level and mRNA level of KIF15 in breast cancer tissues. Then CCK-8, wound healing, transwell and flow cytometry experiments were adopted separately to test cell viability, migration, invasion and cell cycle distribution. We discovered that KIF15 was highly expressed in breast cancer tissues and high level KIF15 was associated with a low survival rate of breast cancer patients. Moreover, silence of KIF15 suppressed cell viability, migration, invasion and cell cycle distribution. Following, we discovered that ZNF367 was the upstream transcription factor of KIF15. In addition, silenced ZNF367 could also repress the growth of breast cancer cells. And rescue experiments indicated that overexpressed KIF15 could counteract the inhibition effect of silencing ZNF367 on the progression of breast cancer. Importantly, we discovered that KIF15 and ZNF367 were associated with the regulation of cell cycle. In short, ZNF367-activated KIF15 accelerated the progression of breast cancer by regulating cell cycle progress.

## Introduction

Breast cancer (BC) is a malignant tumor seriously threatens the health of women in the whole world 1,2. In recent years, the incidence of breast cancer is increasing. Through the development of more than ten years, the early screening technology and treatment of breast cancer are becoming more and more mature. Nevertheless, the overall prognosis for advanced patients is not satisfactory 3,4. Thus, an in-depth investigation of the pathogenesis of BC may conduce to provide a new idea for curing breast cancer.

In recent years, the regulation of mitosis has been researched by plenty of scholars. An increasing number of researches indicate that mitotic inhibitors for microtubules have been explored for treating assorted cancers 5. What's more, the functions of kinesin motor proteins in regulating mitosis have also been studied.

The kinesin superfamily proteins (KIFs) are the kind of conservative motor proteins. Importantly, they can drive microtubule-dependent plus-end motion 6. It is reported that KIFs take part in the crucial cellular process like mitosis and meiosis 7. And a flow of researchers has indicated that KIFs exert the vital functions in the progression of cancer 8. Some of them may regulate the growth of cancer cells, while the others may be associated with drug resistance in lots of cancers.

KIF15 is a tetrameric spindle motor which exert the crucial functions in several cancers. For example, KIF15 is reported that it can accelerate cell growth of bladder cancer through MEK-ERK signaling pathway 9. Dihydropyrazole and dihydropyrrole structures-based design of KIF15 inhibitors are considered as new therapeutic agents for cancer 10. Moreover, overexpressed KIF15 predicts the bad prognosis in lung adenocarcinoma patients 11. In addition, it is also reported that KIF15 is closely associated with the process of cell cycle regulation 12. Nevertheless, the specific functions and mechanisms of KIF15 in BC are not yet elucidated.

In our research, we aimed to investigate the functions and mechanisms of KIF15 in BC, which may offer a new idea for curing BC.

## Materials and methods

### Tissue samples

The ethical approval for our study was acquired from the Ethics Committee of the First Affiliated Hospital, Sun Yat-Sen University, and the informed consents were provided by all patients. A total of 126 females with breast cancer who diagnosed at the First Affiliated Hospital, Sun Yat-Sen University were enrolled in this study. Among them, 36 pairs of breast cancer tissues and para-cancerous tissues were collected for qRT-PCR analysis while 90 breast cancer tissues were subjected to the IHC assay. All participants didn't receive chemotherapy and radiotherapy before surgery.

### Cell lines

Human BC cell lines MDA-MB-231 and SKBR3 were available from the American Type Culture Collection (ATCC; Rockville, Maryland) for this study. Both were grown in Dulbecco's Modified Eagle's medium (DMEM) as instructed by supplier (Invitrogen, Carlsbad, CA), with 10% fetal bovine serum (FBS) and 1% penicillin-streptomycin as supplements. Cell culture was accomplished in a humidified incubator containing 5% CO_2_ at 37 °C.

### Immunohistochemistry

IHC staining was performed to evaluate the expression pattern of ZNF367 in breast cancer tissues. Briefly, slides were dewaxed rehydrated using xylol and a descending alcohol series. Then, endogenous peroxidase activity was blocked at room temperature by incubation in the final development of 3% H_2_O_2_ in distilled water or PBS (pH 7.4) for about 5-10 minutes. The sections were placed in a 0.1 M citrate buffer, pH 5.0, with microwave treatment for 8 minutes, and then cooled to room temperature for antigen retrieval. After blocking with 1% goat serum, the sections were incubated with anti-KIF5 (Proteintech, 55407-1-AP, 1:200) overnight at 4°C. Sections were rinsed twice for 5minutes each and sections incubated with a HRP-conjugated secondary antibody (goat anti-rabbit IgG, KGAA26, 1:1000) at 37°C for 20 minutes. Diaminobenzidine was applied to the sections to produce a brown stain indicating immunoreactivity, and the samples were counterstained with hematoxylin. Images were captured by light microscopy.

### Quantitative real-time polymerase chain action (qRT-PCR)

The total RNA was extracted from cultured MDA-MB-231 and SKBR3 cell samples by employing the RNA extraction kit (Promega, Beijing, China). To evaluate the expression of KIF15 and ZNF367, cDNA was generated via reverse transcription by the use of the PrimeScript™ RT reagent kit (Takara, Shiga, Japan). Then, the qRT-PCR assay was implemented with SYBR Premix Ex Taq II (Takara) on Step-One Plus Detection System Applied Biosystems, Foster City, CA). House-keeping gene GAPDH was utilized as reference gene. Relative gene expression was examined by the 2^-ΔΔCt^ method. Primers for qRT-PCR were shown in Supplementary Table 1.

### Western blot

The total proteins were extracted from cell samples of MDA-MB-231 and SKBR3 in RIPA lysis buffer and then treated with 7.5% SDS-PAGE. The separated cell proteins were shifted onto PVDF membranes and cultured with 5% nonfat milk. After blocking, membranes were probed one night at 4^o^C with the primary antibodies against loading control beta-tubulin and ZNF367, KIF15, CDK2, Cyclin E1, Cyclin E2. Subsequently, the relative secondary antibody was added for 2 h at room temperature. All protein bands were examined by ECL detection system requested (Amersham, Arlington Heights, IL). The information of utilized antibodies was shown in Supplementary Table 2.

### RNA interference and transfection

To stably silence KIF15 and ZNF367, the specific short hairpin RNAs (shRNAs; GeneCopoeia, Maryland, USA) were transfected into cells using Lipofectamine 3000 kit (Invitrogen, along with the nonspecific shRNAs as control. The full-length cDNA sequence of KIF15 was amplified and inserted into pEZ-lv201 vector (GeneCopoeia) for overexpressing KIF15. At 48 h post-transfection, cells were reaped. Sequences for siRNAs and shRNAs were listed in Supplementary Table 3 and the information of vector for shZNF367 and ov-KIF15 was showed in Supplementary Fig 1 and Supplementary Fig 2.

### Cell counting kit-8 (CCK-8)

After transfection, 1 × 10^3^ cell samples at logarithmic growth phase were seeded to each well of 96-well plate for cell viability assay in line with the protocol of CCK-8 kit (Dojindo Laboratories, Kumamoto, Japan). 10 μl of CCK-8 was added for 2 h and then the optical density (OD) at 450 nm was monitored by microplate reader at the time of each 24 hours until 120 hours.

### Wound healing

MDA-MB-231 and SKBR3 cells were cultured for 24 h in serum-free medium and then wounds were generated by pipette tips. The medium was refreshed with FBS-free DMEM, and the distance of wound healing at 0 h and 48 h was separately observed by microscope (Olympus, Tokyo, Japan).

### Transwell invasion assay

Cell samples in serum-free medium were plated into the upper chamber of 8-mm pore size Transwell inserts (Corning, NY) containing Matrigel, and 100% culture medium was applied to supplement the lower chamber. Cells remaining in the top were removed by cotton swabs, while cells migrating to the bottom were fixed by 4 % paraformaldehyde and then counted visually via 0.1% crystal violet dye. Cell migration was determined by counting 5 random fields under microscope.

### Cell cycle assay

The processed cell samples were seeded to 6-well plates with 3 × 10^5^ cells per well, then rinsed in prepared phosphate buffer saline (PBS). After incubation with the 1 mg/ml of propidium iodide (PI; Sigma Aldrich, St. Louis, MI), flow cytometry was applied for the cell population G0/G1, S, and G2/M phases of cell cycle, as guided by supplier (FlowCount, Beckman Coulter, CA, USA).

### Luciferase reporter assay

KIF15 promoter region was amplified and then subcloned into the pGL3-Basic vector (Promega, Madison, WI) for luciferase assay. Cell samples were co-transfected with pGL3 vector and indicated transfection plasmids for 48 h, then subjected to Luciferase Reporter Assay System (Promega). Sequence of Promoter of KIF15 and ZNF367 was shown in Supplementary Table 4. To further clarify the transcriptional regulation of KIF15 by ZNF367, the wild-type or mutant (MUT) binding site of KIF15 were synthesized for the Luciferase reporter assay. The information of luciferase reporter plasmid of KIF15 promoter and ZNF367 overexpressed plasmid were shown in Supplementary Table 5.

### Chromatin immunoprecipitation (ChIP)

The crosslinking reaction was terminated by glycine in MDA-MB-231 cells treated with 1% formaldehyde. The cell lysate was placed in the ultrasonic crushing apparatus to produce 100-1000bp DNA fragments. The anti-ZNF367 (Abcam, ab108141), RNA polymerase II and IgG were added to form the antibody-target protein-DNA complex. After washing and reversing the crss-links, the enriched DNA was purified and then measured by qRT-PCR. The primer sequences were listed in Supplementary Table 6.

### Bioinformatics analyses

Expression profiles of KIF15 and ZNF367 in breast cancer were acquired from the TCGA database of GEPIA (http://gepia.cancer-pku.cn/) 13. Overall Survival (OS) and relapse free survival (RFS) of breast cancer patients for KIF15 and ZNF367 were done using KM plotter (http://kmplot.com). Immunohistochemistry images of KIFs in normal breast tissue from the GTEx project and tumor tissue from TCGA-BRCA were obtained from the human protein atlas (https://www.proteinatlas.org). Genes co-expressed with KIF15 and ZNF367 were screened out from TCGA-BRCA dataset and analyzed with R2: Genomics Analysis and Visualization Platform (http://r2.amc.nl). Based on the data of TCGA BRCA dataset, the RNA expression correlation between KIF15 and ZNF367 was analyzed by Pearson correlation test. Gene Ontology (GO; www.geneontology.org/) analysis, Kyoto Encyclopedia of Genes and Genomes (KEGG; www.genome.jp/kegg/) analysis were separately performed. Both GO and KEGG analyses were carried out and plotted using RStudio software Version 1.1.463 and R package “ClusterProfiler” 14.

### Statistical analysis

Bio-triple was applied for all assays, and experimental results were given as the means ± standard deviation (SD). Data were processed with the SPSS version 19.0 software (IBM Corp., Armonk, NY), and gene linear correlation was analyzed by Pearson's method. The p-value below 0.05 was considered as the threshold of significant statistics by means of Student's t test or one-way/two-way ANOVA.

## Results

### KIF15 was highly expressed in BC tissues

**Figure 1:**
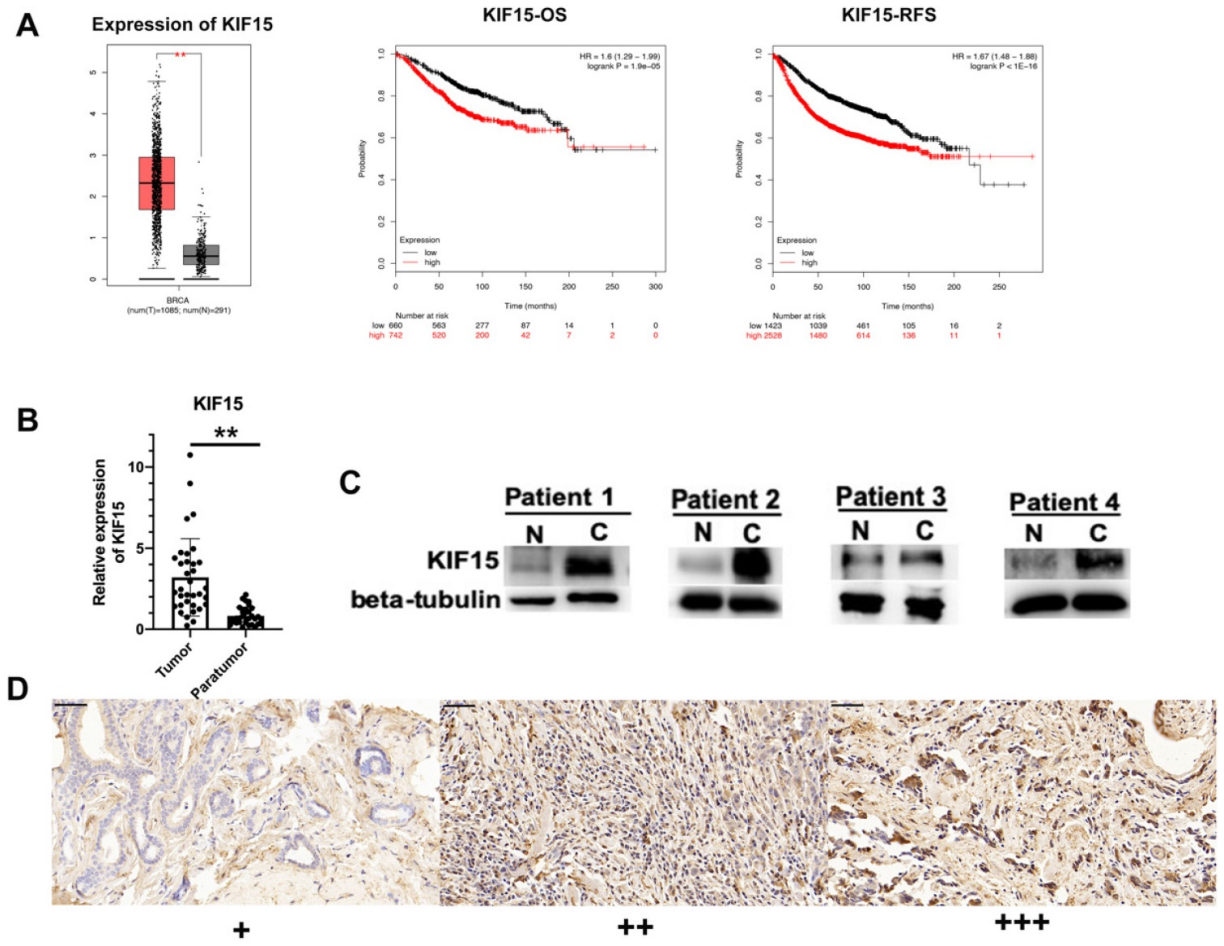
** KIF15 is highly expressed in breast cancer tissues.** (A) TCGA data displayed the expression of KIF15 in BC tissues and normal tissues (left). The overall survival rate, as well as the relapse free survival rate of breast cancer patients with different level of KIF15, was analyzed. (B) The expression of KIF15 in tumor tissues and paratumor tissues of breast cancer. (C) Western blot experiment detected the protein levels of KIF15 in breast cancer tissues. (D) Representative of expression of KIF15 in breast cancer (Original magnification, x400, scale bar 50um). Data displayed in all bar graphs were obtained from three or more independent experiments. ^**^P < 0.01 indicated data were statistically significant.

According to the data of online database GEPIA (http://gepia.cancer-pku.cn/), KIF15 was highly expressed in BRCA samples compared with normal samples (**Fig. 1A, left**). Then, Kaplan-Meier plotter (http://kmplot.com/) was applied to assess the correlation between the level of KIF15 and the overall survival (OS) or the relapse free survival (RFS) of BC patients. Intriguingly, high level of KIF15 indicated the low OS rate and RFS rate in BC patients (**Fig. 1A, middle and right**). To further validate the dysregulation of KIF15 in BC samples, qRT-PCR examination was performed to detect the level of KIF15 in tumor tissues and paratumor tissues collected from 36 patients with BC. Consistently, a higher level of KIF15 was examined in tumor tissues (**Fig. 1B**). Through western blot analysis, the protein level of KIF15 was found to be higher in tumor tissues than that in paratumor tissues (**Fig. 1C**). To identify the protein expression of KIF15 in breast cancer tissue samples, KIF15 was detected by IHC in 90 breast cancer specimens. KIF15 was expressed in cytoplasm and occasionally at nucleus in breast cancer cells (Fig. 1D). The weak staining (+) was found in 19 cases, moderate staining (++) in 37 cases and strong staining (+++) in 34 cases, there was no negative staining in our tissues. To further analysis the relationship between the expression of KIF15 and clinical-pathological characteristics of breast cancer (**Table 1**), we defined weak and moderate staining as normal-expression and strong staining as over-expression. Overexpression of KIF15 in breast cancer was significantly correlated with higher WHO grade (P<0.0001) and higher Ki-67 level (P=0.002). Overall, these experimental data demonstrated that KIF15 was highly expressed in BC patients and predicted poor prognosis.

### Knockdown of KIF15 repressed the migration of BC cells

**Figure 2:**
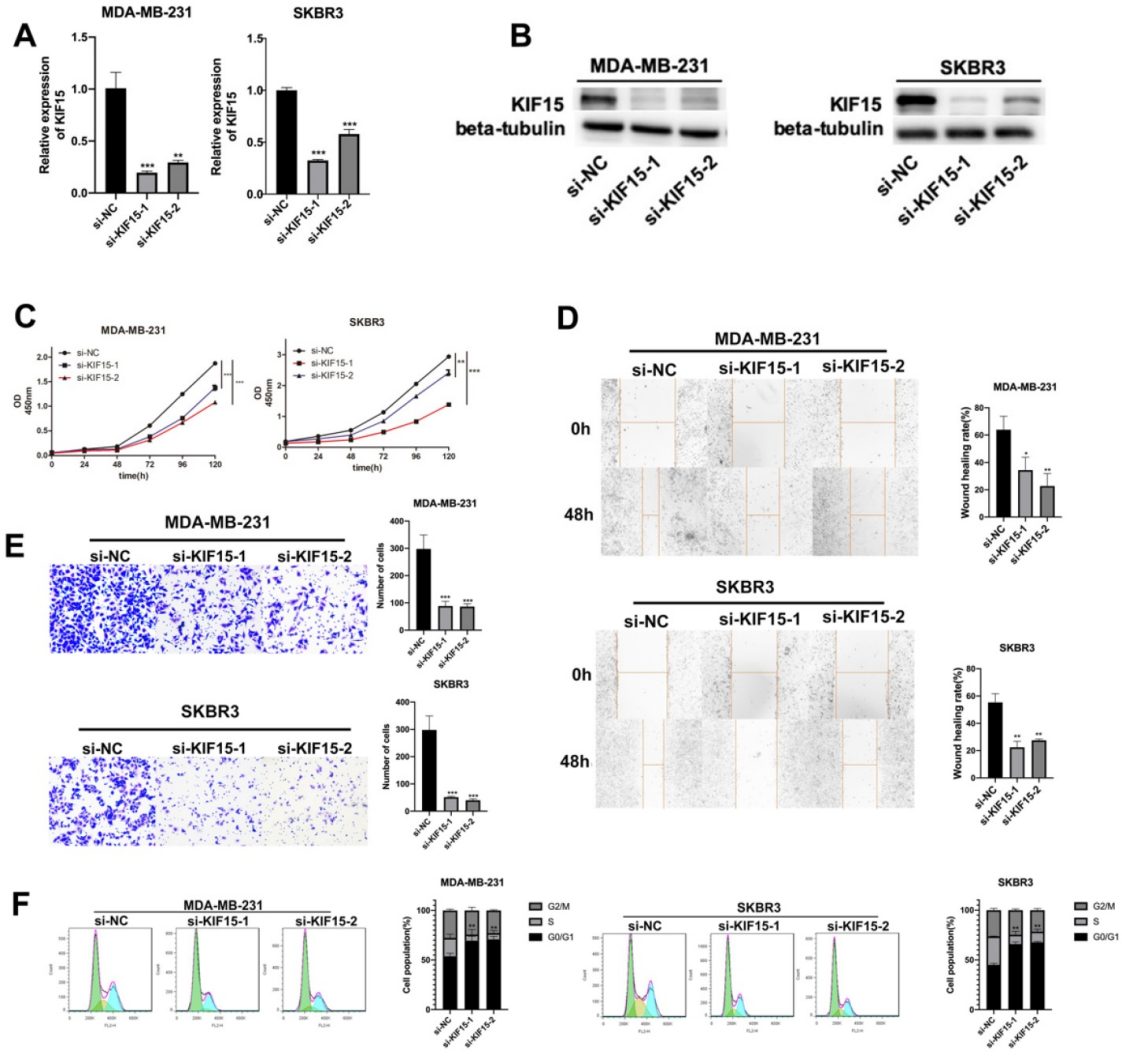
** Knockdown of KIF15 repressed the growth of breast cancer cells.** (A-B) Knockdown efficiency of KIF15 was evaluated in MDA-MB-231 and SKBR3 cells through RT-qPCR and western blot. (C) Cell viability was measured by CCK-8 when KIF15 was inhibited. (D) Wound healing assay was utilized to measure cell migration capability after silencing KIF15. (E) Transwell experiment was conducted to test cell invasion after KIF15 depletion. (F) Flow cytometry was carried out to evaluate the influence of silencing KIF15 on cell cycle. Data displayed in all bar graphs were obtained from three or more independent experiments. ^*^P < 0.05, ^**^P < 0.01,^ ***^P < 0.001 indicated data were statistically significant.

To investigate the role of KIF15 in regulating BC cell functions, loss-of function assays were designed and performed in two BC cells (MDA-MB-231 and SKBR3). At first, KIF15 was efficiently silenced in two BC cells with specific siRNAs targeting KIF15 (**Fig. 2A**). Non-targeted siRNA was used as negative control (si-NC). And western blot displayed that the protein level of KIF15 was also declined in MDA-MB-231 and SKBR3 cells transfected with KIF15-specific siRNAs (**Fig. 2B**). Then, we implemented several functional experiments to evaluate the influence of KIF15 silencing on the biological behaviors of BC cells. CCK-8 experiment was utilized to test cell viability, and the results revealed that cell viability was restrained when KIF15 was inhibited (**Fig. 2C**). Then wound healing assay was implemented to measure cell migration capability. The results indicated that the wound healing rate was declined in MDA-MB-231 and SKBR3 cells after silencing KIF15, indicating that cell migration could be hampered by KIF15 depletion (**Fig. 2D**). As for the capability of cell invasion, we carried out transwell assays. The results depicted that the number of invaded cells was distinctly reduced when KIF15 was knocked down (**Fig. 2E**). In the end, we implemented flow cytometry analysis to evaluate the effect of silenced KIF15 on cell cycle distribution. We discovered that the cell population of G0/G1 phase was elevated when KIF15 was knocked down, while the cell population of S phase was reduced (**Fig. 2F**). It demonstrated that knockdown of KIF15 led to the cell cycle arrested at G0/G1 phase. In short, knockdown of KIF15 repressed the malignant behaviors of BC cells.

### ZNF367 acted as a transcription activator for KIF15

**Figure 3:**
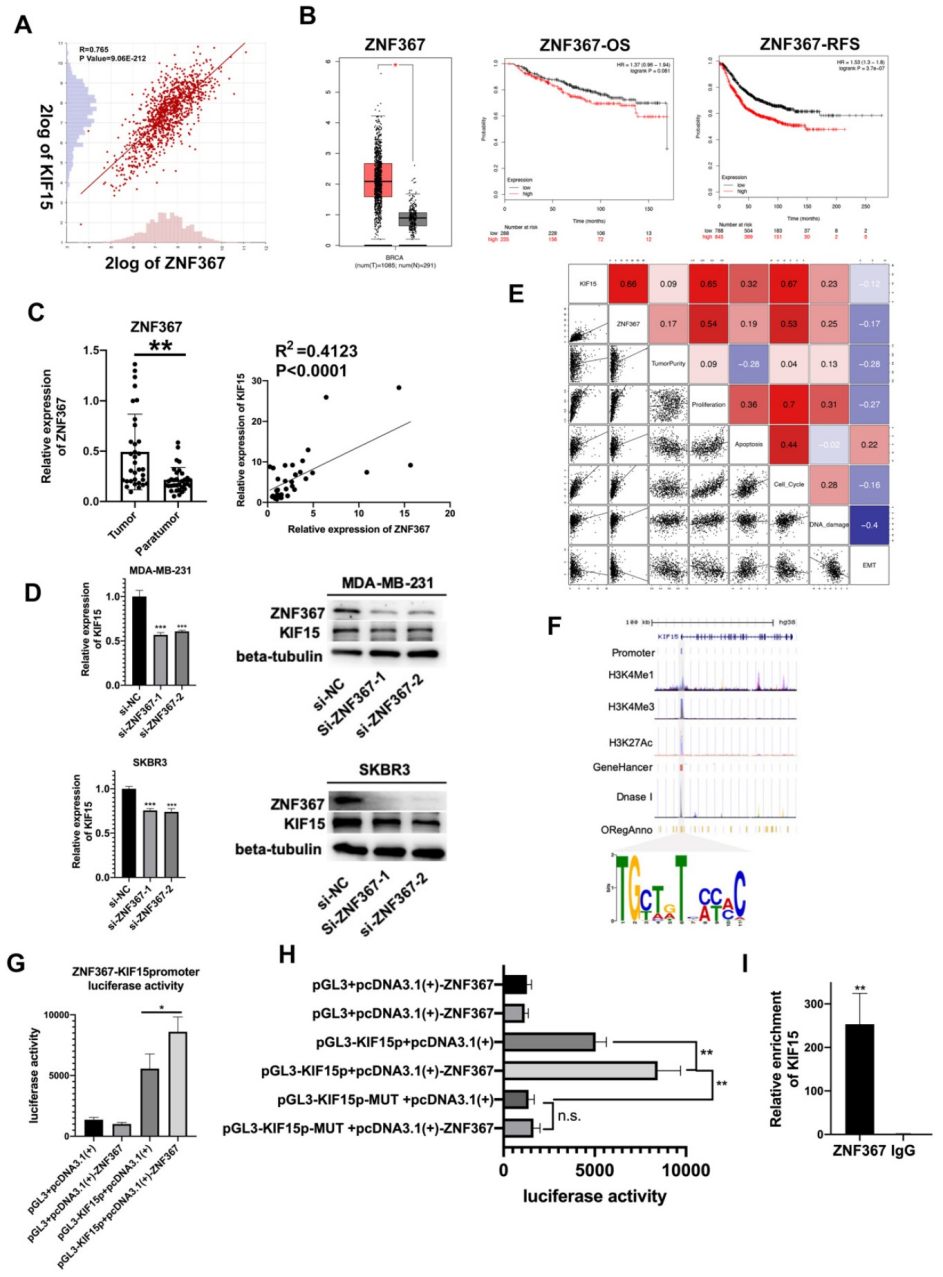
** ZNF367 acts as transcription factor and activates KIF15 expression.** (A) Correlation analysis chart of KIF15 and ZNF367. (B) TCGA data displayed the expression of ZNF367 in breast cancer tissues and the overall survival rate as well as the relapse free survival rate of breast cancer patients with different level of ZNF367. (C) The expression of ZNF367 in tumor tissues and paratumor tissues of breast cancer. And the correlation analysis chart of KIF15 and ZNF367. (D) Western blot experiment tested the influence of ZNF367 knockdown on the mRNA and protein levels of KIF15. (E) There was a strong correlation between the cellular functions of KIF15 and ZNF367. (F) DNA motif and the enrichment of H3K4Me1, H3K4Me3 and H3K27Ac of KIF15 promoter were predicted and analyzed. (G) Luciferase reporter analysis revealed the luciferase activity of KIF15 promoter in response to the overexpression of ZNF367. (H) The wild-type, or mutant (MUT), binding site of KIF15 was synthesized for the Luciferase reporter assay. (I) ChIP-qPCR analysis demonstrated that ZNF367 could bind to the promoter of KIF15 gene and strengthen its transcription activity. Data displayed in all bar graphs were obtained from three or more independent experiments. *P < 0.05, **P < 0.01, ***P < 0.001 indicated data were statistically significant.

Upstream transcription factors of KIF15 were predicted and explored. According to online dataset and Pearson correlation test, ZNF367 was found to be positively correlated with KIF15 in BC tissues in mRNA level (**Fig. 3A**). Consistent with KIF15, ZNF367 was also highly expressed in TCGA BRCA patient samples (**Fig. 3B, left**). And overall survival rate and relapse free survival rate were low in BC patients with high level ZNF367 (**Fig. 3B, middle and right**). Following, we discovered that ZNF367 level was higher in 36 tumor tissues than that in paratumor tissues (**Fig. 3C, left**). And then the correlation analysis manifested the positive correlation between ZNF367 and KIF15 (R^2^=0.4123, P<0.0001) (**Fig. 3C, right**). Then, we utilized the siRNAs targeting ZNF367 to silence ZNF367 expression in MDA-MB-231 and SKBR3 cells and implemented western blot to test the influence of silencing ZNF367 on the protein levels of ZNF367 and KIF15. Results indicated that the expression of KIF15 could be reduced by ZNF367 depletion both in mRNA and protein levels (**Fig. 3D**). Functional enrichment results indicated that there was a strong correlation between the cellular functions of KIF15 and ZNF367 (**Fig. 3E**). We found that the modification of KIF15 gene mainly occurred in the promoter region, and the motif of ZNF367 annotated in the database was matched with the transcriptional regulate motif of KIF15 in the promoter region. Thus, ZNF367 may bind to the promoter region of KIF15 (**Fig. 3F**). Finally, luciferase reporter assay was conducted to estimate whether ZNF367 could bind to KIF15 promoter region. We discovered that overexpressing ZNF367 enhanced the luciferase activity of KIF15 promoter (**Fig. 3G**). To further clarify the transcriptional regulation of KIF15 by ZNF367, the wild-type or mutant (MUT) binding site of KIF15 were synthesized for the Luciferase reporter assay. The result showed that ZNF367 promoted the luciferase activity of wild type KIF15 promoter but not mutant KIF15 promoter (**Fig. 3H**). Besides, ChIP-qPCR analysis demonstrated that ZNF367 could bind to the promoter of KIF15 gene and enhance its transcription activity (**Fig. 3I**). Overall, ZNF367 positively regulated KIF15 through transcriptionally activating KIF15 in breast cancer.

### Silenced ZNF367 restrained BC cellular processes

**Figure 4:**
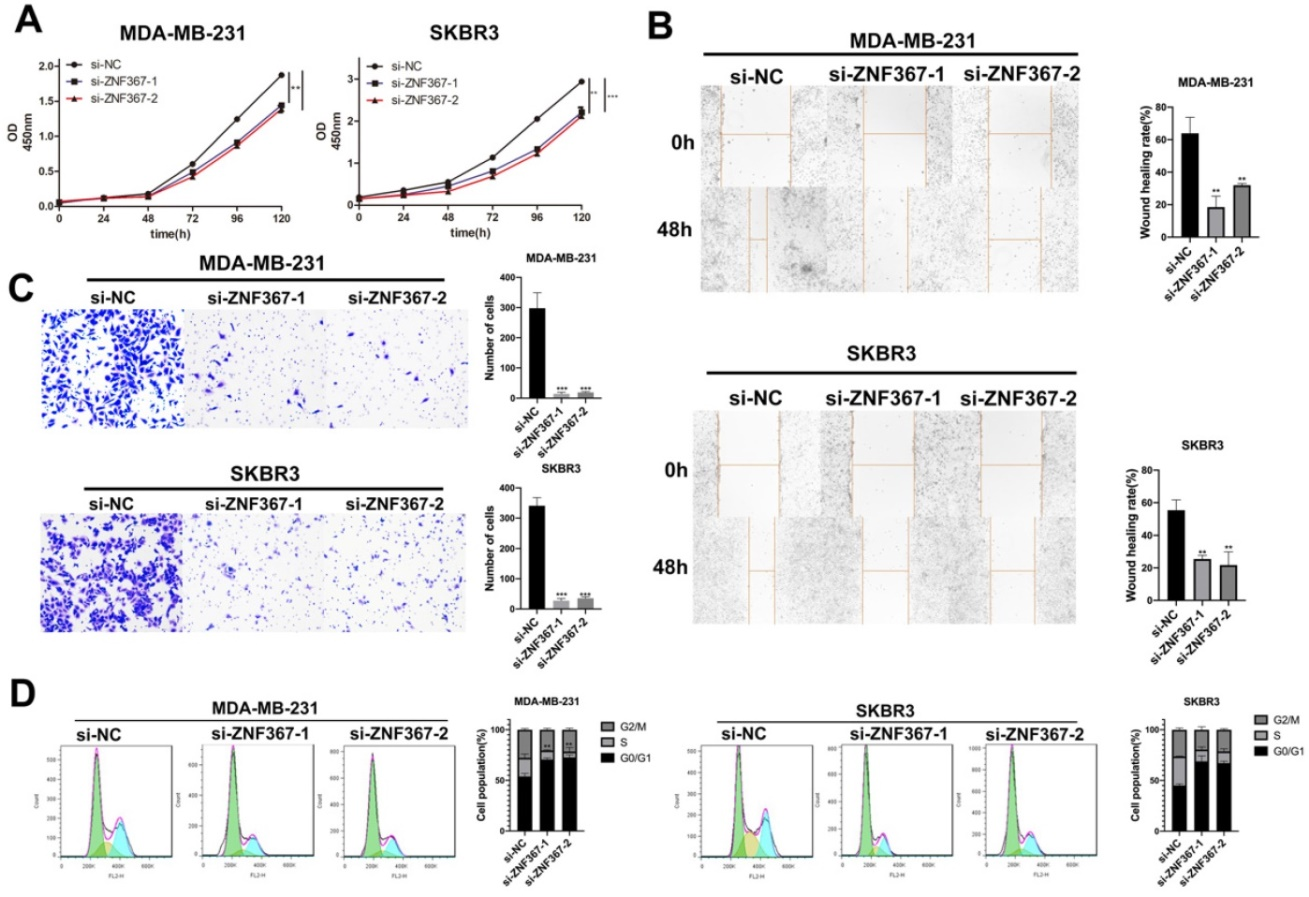
** Silenced ZNF367 restrains cell growth of breast cancer.** (A) CCK-8 assay was implemented to estimate cell viability when ZNF367 was subjected to knock down. (B-C) Wound healing assay and transwell assay were utilized to test cell migration and invasion after silencing ZNF367. (D) Flow cytometry detected the influence of inhibiting ZNF367 on cell cycle. Data displayed in all bar graphs were obtained from three or more independent experiments. ^**^P < 0.01,^ ***^P < 0.001 indicated data were statistically significant.

Next, we carried out a series of functional experiments to evaluate the functions of ZNF367 in BC cells. Though CCK-8 assay, we knew that cell viability of MDA-MB-231 and SKBR3 was hampered when inhibition of ZNF367 expression (**Fig. 4A**). Wound healing assay displayed that the wound healing rate was declined in MDA-MB-231 and SKBR3 cells, indicating that cell migration ability was restrained by ZNF367 deficiency (**Fig. 4B**). In addition, it was also depicted through transwell experiments that the number of invaded cells was reduced, which indicated that ZNF367 deficiency weakened cell invasion capability (**Fig. 4C**). Moreover, flow cytometry analysis was conducted and we discovered that G0/G1 phase was increased and S phase was reduced, demonstrating the arrest of cell cycle by ZNF367 knockdown (**Fig. 4D**). Taken together, silencing of ZNF367 restrained cell growth and migration in breast cancer.

### ZNF367 transcriptionally activated KIF15 and regulates cell cycle in breast cancer

**Figure 5:**
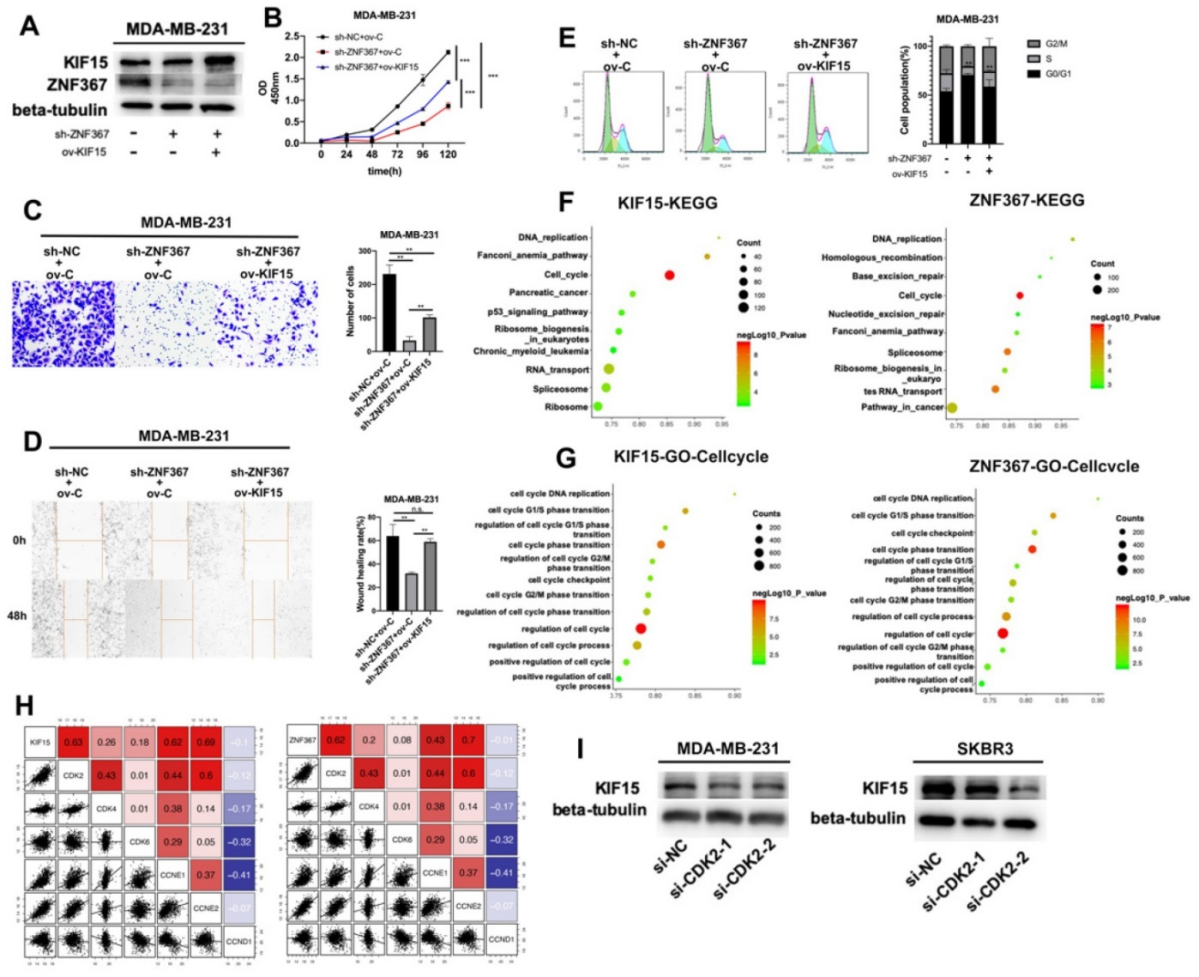
** ZNF367 transcriptionally activates KIF15 and regulates cell cycle in breast cancer.** (A) Western blot experiment was utilized to test the protein level of ZNF367 when ZNF367 was silenced and the protein level of KIF15 when KIF15 was overexpressed. (B) CCK-8 experiments detected the influence of the interaction between KIF15 and ZNF367 on cell viability. (C-D) Wound healing assay and transwell assay measured the capabilities of cell migration and invasion when ZNF367 was silenced and KIF15 was overexpressed. (E) Flow cytometry detected cell cycle after silencing ZNF367 and upregulating KIF15. (F-G) KEGG signaling pathway analysis and GO-Cell cycle analysis were conducted to detect the function of KIF15 and ZNF367. (H) Correlation analysis between KIF15 or ZNF367 and G1/S phrase cycle-related proteins (CDK2, CDK4, CDK6, CCNE1, CCNE2 and CCND1). (I) Western blot experiment evaluated the influence of silencing CDK2 on the protein level of KIF15. Data displayed in all bar graphs were obtained from three or more independent experiments. ^**^P < 0.01,^ ***^P < 0.001 indicated data were statistically significant. “n.s.” indicates no significance.

Finally, rescue experiments were conducted in MDA-MB-231 cells to verify the role of the ZNF367/KIF15 axis in BC cell functions. First of all, western blot assay detected the protein level of KIF15 and ZNF367 in transfected cells and we discovered that the decreased level of KIF15 protein was recovered after overexpression of KIF15. However, the level of ZNF367 protein decreased by sh-ZNF367 was not changed after overexpression of KIF15 (**Fig. 5A**). Then CCK-8, transwell and wound healing assays were carried out for evaluating the influence on cell viability, invasion and migration. We discovered that cell viability was weakened by ZNF367 knockdown but then reversed by KIF15 overexpression (**Fig. 5B**). And impaired cell migration and invasion capabilities caused by the lack of ZNF367 were recovered by the upregulation of KIF15 (**Fig. 5C-D**). What's more, flow cytometry experiment was conducted to measure cell cycle. Similarly, overexpressed KIF15 could offset the inhibited effect of silenced ZNF367 on cell cycle progress (**Fig. 5E**). Taken together, ZNF367 transcriptionally activated KIF15 and accelerated the progression of breast cancer. Thereupon, we conjectured KIF15 and ZNF367 were associated with the progression of cell cycle. Genes that were co-expressed with KIF15 or ZNF367 were screened out from TCGA-BRCA data using R2: Genomics Analysis and Visualization Platform (http://r2.amc.nl). Subsequently, these co-expressed genes were subjected to KEGG and GO enrichment analyses with RStudio software Version 1.1.463 and R package “ClusterProfiler”. Accordingly, KEGG signaling pathway analysis revealed that both of KIF15 and ZNF367 have the highest correlation coefficient with cell cycle (**Fig. 5F**). According to the results of GO analysis, KIF15 and ZNF367 signals were significantly correlated with cell cycle progress (**Fig. 5G**). In addition, we further conducted the correlation analysis between G1/S phrase cycle-related proteins (CDK2, CDK4, CDK6, CCNE1, CCNE2 and CCND1) and KIF15 or ZNF367, and the results displayed that KIF15 and ZNF367 have the most significant correlation with CDK2 (**Fig. 5H**). Thus, we knocked down CDK2 in MDA-MB-231 and SKBR3 cells to measure the protein levels of KIF15 through western blot experiments, and we found that the protein level was decreased by the knockdown of CDK2 (**Fig. 5I**). Moreover, the protein levels of ZNF367, Cyclin E1 and Cyclin E2 were detected with western blot assay and the results showed that the levels of all these proteins were not significantly changed after silencing of CDK2 (**Supplementary Fig. 1**). Consistently, downregulation of ZNF367 had no significant effect on the level of CDK2 protein (**Supplementary Fig. 2**). In short, these results demonstrated that ZNF367 and KIF15 could regulate cell cycle in breast cancer.

## Discussion

Kinesin superfamily proteins (KIFs) are a kind of conservative motor proteins. In different stage of mitosis, lots of kinesins exert crucial functions in cell division. In recent years, increasing number of reports has confirmed KIFs as the key regulatory factors in cancer development, including breast cancer. For example, KIF-2C was considered to be associated with the poor prognosis of male patients with esophageal squamous cell carcinoma. The prognosis of patients with high level KIF-2C was worse than others 15. It was reported that down-regulation of KIF11 decreased the proliferation of meningioma cells 16. Accordingly, KIF11 was supposed to be the novel therapeutic target of meningioma. Moreover, KIF23 was highly expressed in gastric cancer cells and it accelerated the progression of gastric cancer 17. In our research, we investigated the function of KIF15 in breast cancer. Just like other members of KIFs family, KIF15 was also proven to exert the vital functions in human cancers, such as pancreatic cancer 18, bladder cancer 19 and hepatocellular carcinoma 20. KIF15 expression in those cancers was extremely high and it accelerated cancer progression. In our study, we discovered that KIF15 expression was upregulated in BC tissues in comparison with normal tissues. Importantly, we knocked down KIF15 in BC cells and demonstrated that silencing KIF15 restrained cell viability, migration, invasion and cycle, indicating that KIF15 exert the carcinogenic effect in BC.

Transcription factors have also been intensively studied in recent years. They have been confirmed to regulate gene transcription and affect genes expressions. For example, upregulation of SOX2 activated PVT1 expression and accelerated the progression of breast cancer 21. And RGMB-AS1 was activated via E2F1 and expedited migration and invasion in papillary thyroid carcinoma cells 22. ZNF367 was a transcription factor and we found ZNF367 was one of the most significant upstream transcription factors of KIF15. What's more, we proved that ZNF367 was positively correlated with KIF15 and transcriptionally activated KIF15. In addition, functional experiments demonstrated that silenced ZNF367 could repress cell growth of breast cancer. Rescue experiments confirmed that overexpressed KIF15 could offset the inhibitory functions of silencing ZNF367 on the BC progression.

Interestingly, we discovered that cell cycle progression was associated with KIF15 and ZNF367. Through our correlation analysis, both of KIF15 and ZNF367 had the most significant correlation with CDK2, which was one of the cycle-related proteins. It was indicated that ZNF367 and KIF15 could regulate cell cycle in breast cancer so as to accelerate the progression of breast cancer. Importantly, it was reported through previous studies that cell cycle distribution mediated by CDK2 could lead to drug resistance 23. Our work proved that KIF15 and ZNF367 could regulate cell cycle and they were the most significantly related to CDK2 protein. Our work may provide a new theoretical basis for reversing the drug resistance of CDK4/6 inhibitors. In future work, we will focus on research in the deep mechanism underlying ZNF367/KIF15 axis.

On the whole, this study uncovered that ZNF367-activated KIF15 accelerated the progression of breast cancer and exerted the vital functions on cell cycle. This work may offer a new idea for the diagnosis and treatment of breast cancer.

## Supplementary Material

Supplementary figures and tables.

## Figures and Tables

**Table 1:** The association between KIF15 expression and clinical-pathological characteristics in breast cancers

Clinical-pathological characteristics	Total case	Over-expression of KIF15	P value
**Age**			0.249
<50	36	11	
≥50	54	23	
**Tumor Size (cm)**			0.895
≤2	59	22	
>2	31	12	
**Lymph-node metastasis**			0.142
Negative	46	14	
Positive	44	20	
**TNM stage**			
I-II	62	23	0.843
III-IV	28	11	
**ER status**			0.859
Negative	36	14	
Positive	54	20	
**PR status**			0.509
Negative	41	17	
Positive	49	17	
**Her-2 expression**			0.925
Negative	71	27	
Positive	19	7	
**Ki-67 status**			0.002**
≤14%	43	9	
>14%	47	25	
**WHO grade**			<0.0001***
I-II	51	10	
III	39	24	

**P<0.001, ***P<0.0001.

## Abbreviations

BC: breast cancer
KIF15: Kinesin superfamily proteins 15
ZNF367: Zinc finger factor 367
siRNA: small interfering RNAs
FBS: fetal bovine serum
CCK-8: cell counting kit-8
qRT-PCR: Quantitative real-time polymerase chain action
OS: overall survival
RFS: relapse free survival
