# Supplementary material for: ZNF367-induced transcriptional activation of KIF15 accelerates the progression of breast cancer
Source: Int J Biol Sci. 2020 May 16;16(12):2084–93. doi: 10.7150/ijbs.44204 (PMC7294947; doi:10.7150/ijbs.44204)
Supplement: Supplementary file 1 — Supplementary figures and tables. [file ijbsv16p2084s1.pdf]

Supplementary Table 1 Primers for qRT-PCR

**ZNF367-F**	TGTGTGACTATCCAGACTGTGG
**ZNF367-R**	TGCATGGGTGAATCTGCTCAG
**KIF15-F**	AGGAATCTGTATTCGCAACTGTG
**KIF15-R**	ACTTCGTGGGATTACTCCTCTC
**GAPDH-F**	GGAGCGAGATCCCTCCAAAAT
**GAPDH-R**	GGCTGTTGTCATACTTCTCATGG

Supplementary Table 2 Information of utilized antibodies

**Antibody**	**Dilution**	**Manufacturer**	**Cat No.**
**KIF15**	1:1000	Proteintech(Wuhan, China)	55407-1-AP,
**ZNF367**	1:2000	Abcam(Cambridge, MA, US)	ab108141
**beta-tubulin**	1:25000	Proteintech(Wuhan, China)	66240-1-Ig
**CDK2**	1:1000	Proteintech(Wuhan, China)	10122-1-Ap
**CyclinE1**	1:1000	Proteintech(Wuhan, China)	11554-1-Ap
**CyclinE2**	1:500	Proteintech(Wuhan, China)	11935-1-Ap

Supplementary Table 3 Sequence for siRNAs and shRNAs

**si-KIF15-1**	CAGCCAUAAUUGCAAAUGU
**si-KIF15-2**	GUGCCCUUUACAACAAAGA
**si-ZNF367-1**	GCACCUUAUUUGCUUAUCA
**si-ZNF367-2**	GCGAGGUAUUGGGAAAUGA
**si-CDK2-1**	GGAGCUUGUUAUCGCAAAU
**si-CDK2-2**	GCCGUACCAAUCUCUGAAU
**sh-ZNF367**	GGACACACTCAGCAAACATCA

Supplementary Table 4 Information of Sequence of Promoter of KIF15 and ZNF367

Sequence of KIF15 promoter:

ccaagtagctgggaccacaggtgcatgccaccacactgggctaaattttgtatttttgtagagatggggtctcaccatgtt
ggccaggctggtcttgaactcctgggctcaagcaatccacctgccttgacttcccaaagtgctgggattacaagtgtgag
ccattgcgcctggctgttatttcttaaataataagactgaaaatagaaattactccttgatccatgggctgcagaatggatgt
tgtgttagcaggcatgaaaaccccacgttaatctccttgtagatctccatcagagttcttgggtgaccaagtgtattgtccat
gagcaataatatattgaaagaatcttttttttctgagcagtaggtctcaacagtgggctaaaaataattcagtaaaccatatt
ataaagagatgtgctgtcatcaaggttttgtttttccatttctagagcacaggcagagtagatttagcataattcttacggacc
ctaggatttttggaatggtaagtattagcttcaacttaaaagtcaccagctgcattagctcctaatgagagagttagcctgtc
ctttgaaaatatgaagccaggcactgacttctctctagctatgaaagtcctaaatggcccaggcatagtggtttttgagag
gctttgagaggctaaagtgggaggattgcttgagtccaggagtttgagaccagccttggcaacacagtggaatcctgtct
ctacaaaaaaatacaaaaattagccagatgcgttagttcatgcctgtagtcccagctgcttgagaggctgaggcaggag
gatcacttgagcccaggaggtcaaggctgcagtgagctgtgattgcaccactgcactctagcctgggcgacagagtga
gaccctatctcaaaaaaaaaaaaaaaaaaaaagtcctagatggcatcttcttccaatagaaggctgtttcatctacattgaa

aacctgtttagtgtagccaccttcatcaactatcttagctagatctgaataatttgcttcttcatctcatacttttatgttatggag
atggcttctttccttaaacctcatgaaccaacctctgttagcttcacacttttcttctgcagctttctcacatgtctcagacttca
caggatttaagagttagggccttgctctagattaggctttggttaaagggaatgttgtggctggtttgctcttctatccagac
cactaaaactttctctgtatcagcaatgtagctgttttgctttcttgtcattcatgtgttcaatactgatccctccggccctagac
aacaatagtcaggtgtggctctttcctgaagctcctcttcttctatttagacccaggatatttacggcttctgcttactcttctttt
ttttcttttttttgagacggagtctcgctctgtcgcccagtctggagtgcagtggcgcgatctcggctcactgcaagctcctc
ctcctgggttcacgccattctcctgcctcagcctcccgagtagctgggactacaggcgcccgccaccatgcctggctaa
ttttttgtatttttagtagagacgcggtttcactgttttagccaggatggtctcgatctcctgacctcgtgatctgccgcctcag
cctcccagagcgctgggattacaggcgtgagccaccaggcccggcttttttttttttttttgagacggagtctcgctctgtt
gtccaggctggagtgcagctgcgccatctcaactcactgcaacgtccacctcccgggttcgagcgattctgtctcagcct
ccggagtagctgggactacaggcatgtgccaccatgcccggctaatttttgtatttttagcagagacgggggtttcacc
atattggctattggctctgctggtctcgaactcctgacctcaggtgatccgcccacctaggcctcccaaagtactgggatt
acaggcgtgagccactgtgcccgaccttctctatattatttttatttatttttaattaacaaagcatagaaacagattcaggac
attcctccgaagacgttccagactcgagtcctaggacatggttggggcgggggtcatttgatcgagtggcctgtgtcctt
atctggaatacgggacgacgctgtaactctaactcaccctgcgaactcacagcttttgaggtaaacaagtgagaaagaa
gagacaataaagtgatctttctttaccatctaccaaggaccaggtggtttacacgtcagttcattttcaggaacacagcagc
gctctgaaaactacagagcttcctgggcgtagggggctgtggtcgcttgaaaagaaccagcctgggggtccgagttcc
accggaattgggttcgagcgcgggtgaaaagagggacccgacagcacccaccctccaagtagaggcaaaagttgaa
agtggcgggctggctgcgcttccgaagaaacacgactggcgaaggctcaccttagtctctacggtgggtccctccctgt
agccgacccgttccttgaagcgggccagaaacgccggctcggctggccgcacgtacgatacctggttccgcttgctca
tggtagctctgggtaaagacagaagacaggttccgcgacggattcgcgacgatacagaggcgcttagctgtgacgtca
ctgggccacgcccccgatgcgtaggccgggcaacatggcggcctccatgcgggcgtcaacgtccgatccaagcgcc
aaattcaaatttgcggccatcttgagcgggcggaa

Sequence of ZNF367:

CAGAGCGGTGCTCAGGAGTGCGGCCCGGGGCCGCCGGAGCTGGGGTC
GGGCCGGGCGATGATCCGGGGCTTCGAGGCGCCCATGGCGGAGAACCC
GCCGCCGCCGCCGCCCGTCATCTTCTGCCACGACTCCCCGAAGCGG
GTGCTGGTGTCGGTCATCAGGACGACCCCGATCAAGCCAACGTGCGGCG
GTGGAGGGGAGCCGGAGCCGCCGCCGCCGCTCATCCCCACCAGCCCCG
GCTTCAGCGACTTCATGGTGTACCCGTGGCGCTGGGGCGAGAACGCACA
CAACGTGACGCTCAGCCCTGGGGCCGCGGGGGCCGCCGCCTCGGCCGC
CCTGCCTGCAGCCGCAGCCGCCGAGCACTCGGGGCTTCGTGGCCGGGGC
GCGCCCCCGCCCGCCGCCTCGGCCTCCGCCGCCGCCTCGGGAGGTGAGG
ACGAGGAGGAAGCGAGCAGCCCAGACAGCGGCCACCTCAAGGATGGA
ATCCGACGTGGTAGGCCCAGAGCAGATACTGTCCGCGATTTAATAAATG
AAGGAGAGCATTCATCCAGCAGAATCCGTTGTAACATCTGTAATAGGGT
GTTTCCACGGGAGAAATCGCTCCAGGCTCACAAAAGGACTCATACAGGT
GAGAGGCCCTATCTGTGTGACTATCCAGACTGTGGAAAAGCCTTTGTTC
AAAGTGGACAGCTCAAAACACATCAGCGTCTTCACACCGGAGAGAAAC
CTTTTGTTTGTTCAGAAAATGGCTGCCTGAGCAGATTCACCCATGCAAA
CCGCCACTGTCCGAAGCACCCCTACGCCAGGCTGAAGAGAGAGGAGCC
CACGGACACACTCAGCAAACATCAGGCTGCCGACAACAAGGCCGCGGC
CGAGTGGCTGGCGAGGTATTGGGAAATGAGAGAGCAGCGCACCCCCAC

TTTGAAAGGCAAGCTGGTTCAGAAGGCTGATCAGGAGCAGCAGGACCC
TCTGGAATACCTTCAGTCTGATGAAGAGGACGACGAGAAGAGAGGGGC
CCAGCGCCGGCTGCAGGAGCAGCGGGAGCGCCTGCATGGAGCCCTCGC
GCTCATAGAGCTTGCCAACCTGACTGGGGCGCCACTCCGACAGTAGCTT
GGACACTGACTCTTCCACTGTACAAAAGTACTGCCCAGCATACTTAAAA
AGTAGATCCTTGGGCATAAGCTAAGCACC(The coding area was marked red)

Supplementary Table 5 Information of luciferase reporter plasmid of KIF15 promoter and ZNF367 overexpressed plasmid

**ZNF367-F-EcoRI-4**	tatgaattcAGAGCGGTGCTCAGGAGTG
**ZNF367-R-Xho1-4**	tatctcgagGGTGCTTAGCTTATGCCCAAGG
**KIF15promoter-F-Xho1**	Tatctcgag tgctgtcatca aggttttgtttttccatttctagagc
**KIF15promoter-MUT-F-Xho1**	Tatctcgag gttaagctctc aggttttgtttttccatttctagagc
**KIF15promoter-R-HindIII**	tcaAAGCTTcatgttgcccggcctacg

Supplementary Table 6. Information of Primer Sequence for the Chip-qPCR:

GAPDH	F	GGTTTTACGGGCGCACGT
	R	GCTGACTGTCGAACAGGAGG
KIF15	F	GAGCAGTAGGTCTCAACAGTG
	R	ATCTACTCTGCCTGTGCTCTAG

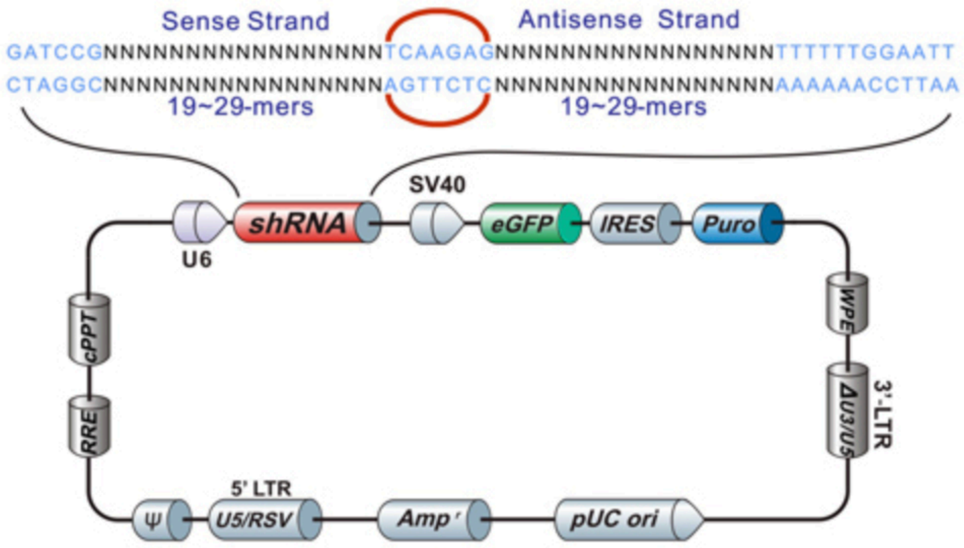

Supplementary Fig. 1 Vector information for shZNF367

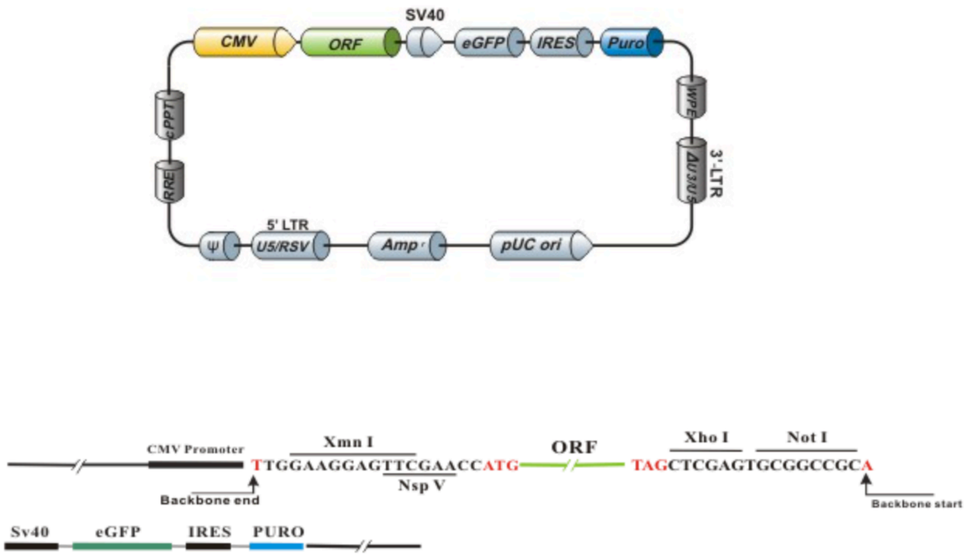

Supplementary Fig. 2 Vector information for KIF15 overexpressed plasmid

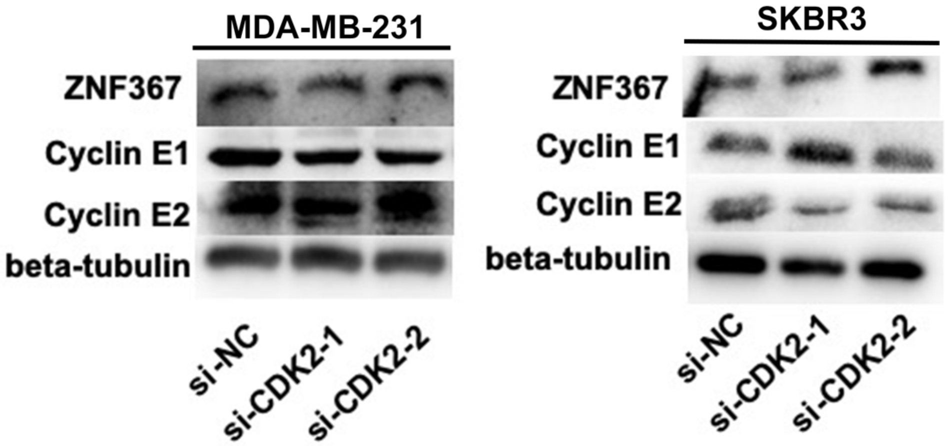

Supplementary Fig. 3 Detection of expression of ZNF367, Cyclin E1, Cyclin E2 by Western blotting assay in the MDA-MB-231 and SKBR3 cell lines after the downregulation of the expression of CDK2.

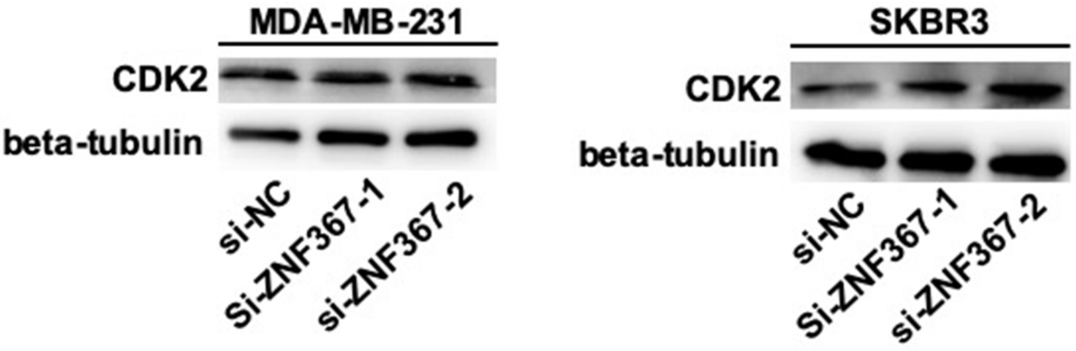

Supplementary Fig. 4. Detection of expression of CDK2 by Western blotting assay in the MDA-MB-231 and SKBR3 cell lines after downregulation of the expression of ZNF367.

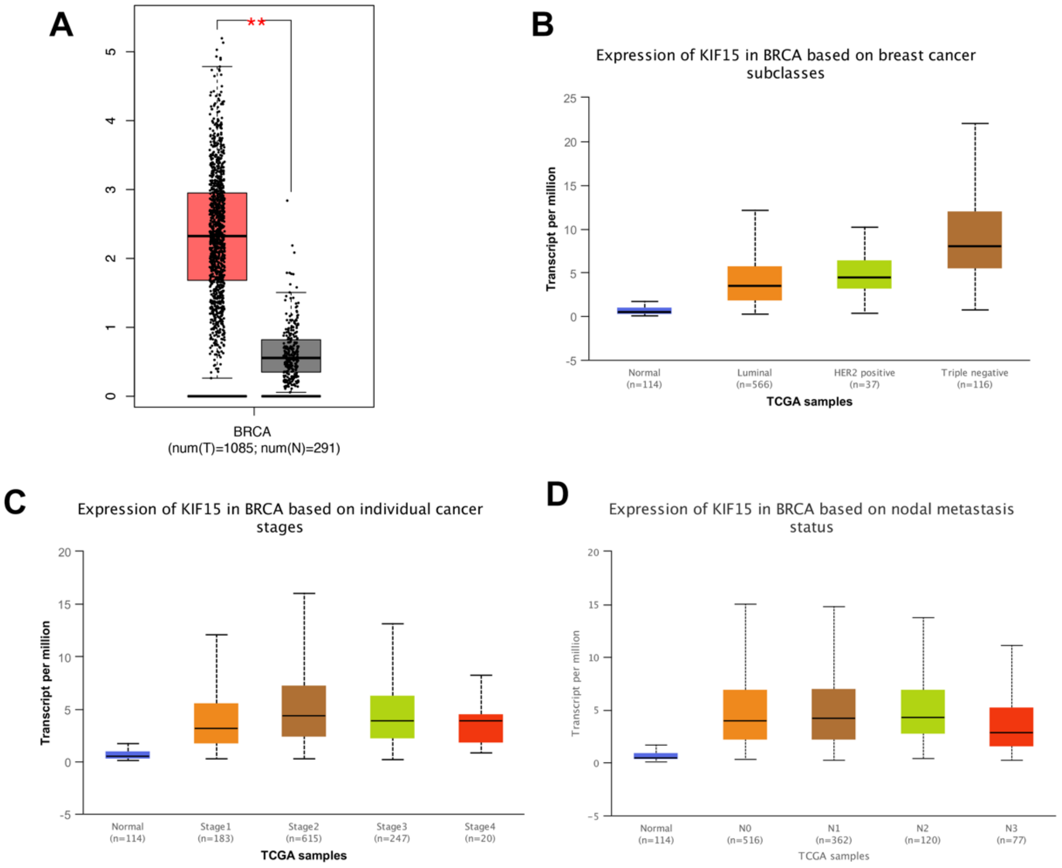

Supplementary Fig. 5 Expression information about KIF15 according to the online database. (A) Expression of KIF15 in BC tissues and normal tissues. (B) Expression of KIF15 in breast cancer subclasses. (C) Expression of KIF15 in individual cancer stages of breast cancer. (D) Expression of KIF15 in different nodal metastasis status of breast cancer.

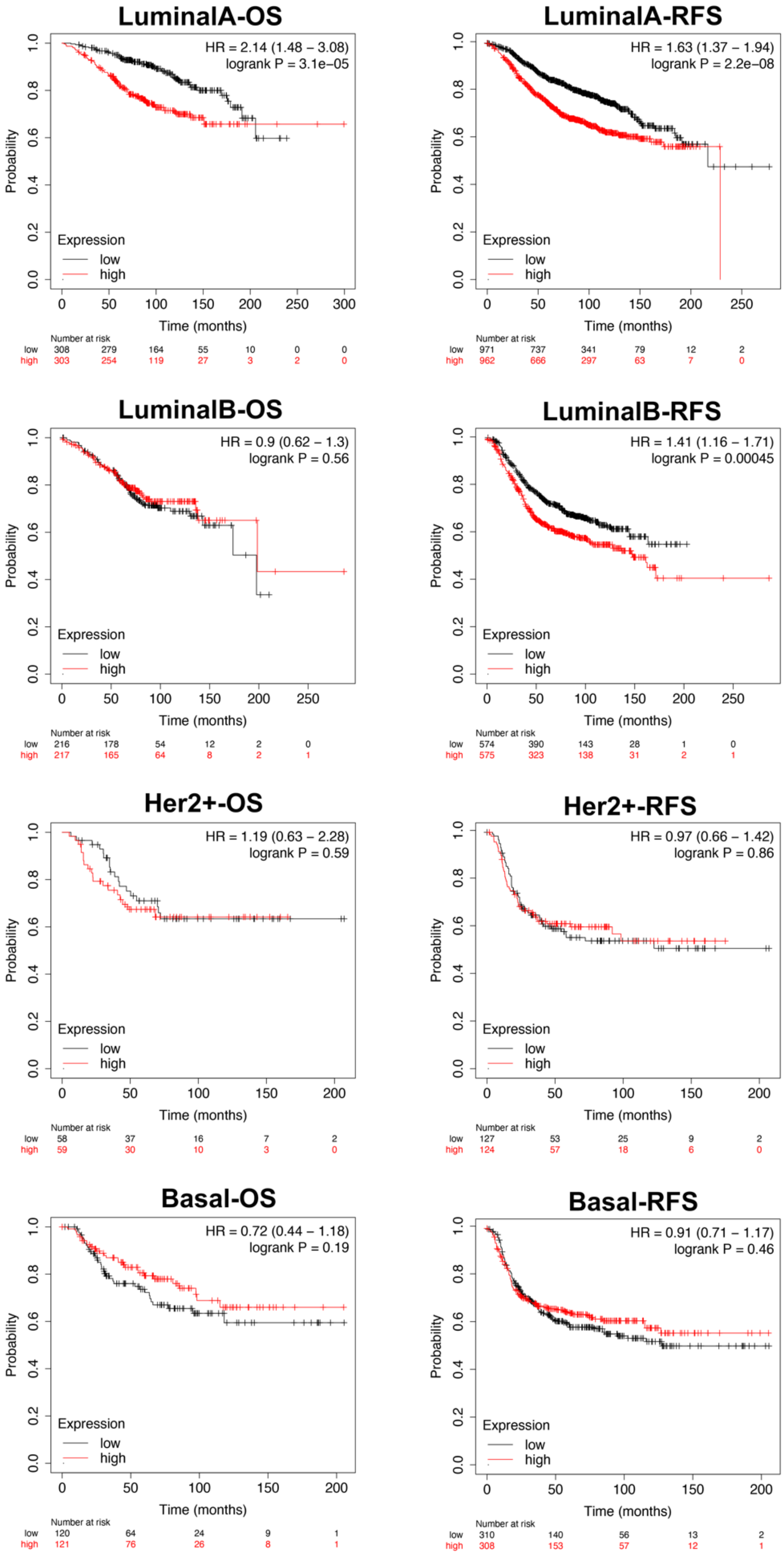
LuminalA-OS
HR = 2.14 (1.48 – 3.08)
logrank P = 3.1e−05
Expression
low
high
Probability
Time (months)
Number at risk
low 308 279 164 55 10 0 0
high 303 254 119 27 3 2 0
LuminalA-RFS
HR = 1.63 (1.37 – 1.94)
logrank P = 2.2e−08
Number at risk
low 971 737 341 79 12 2
high 962 666 297 63 7 0
LuminalB-OS
HR = 0.9 (0.62 – 1.3)
logrank P = 0.56
Number at risk
low 216 178 54 12 2 0
high 217 165 64 8 2 1
LuminalB-RFS
HR = 1.41 (1.16 – 1.71)
logrank P = 0.00045
Number at risk
low 574 390 143 28 1 0
high 575 323 138 31 2 1
Her2+-OS
HR = 1.19 (0.63 – 2.28)
logrank P = 0.59
Number at risk
low 58 37 16 7 2
high 59 30 10 3 0
Her2+-RFS
HR = 0.97 (0.66 – 1.42)
logrank P = 0.86
Number at risk
low 127 53 25 9 2
high 124 57 18 6 0
Basal-OS
HR = 0.72 (0.44 – 1.18)
logrank P = 0.19
Number at risk
low 120 64 24 9 1
high 121 76 26 8 1
Basal-RFS
HR = 0.91 (0.71 – 1.17)
logrank P = 0.46
Number at risk
low 310 140 56 13 2
high 308 153 57 12 1

Supplementary Fig. 6 The overall survival rate as well as the relapse free survival rate of breast cancer patients with different level of KIF15 were analyzed.
